# Transfer Learning Approach with Features Block Selection via Genetic Algorithm for High-Imbalance and Multi-Label Classification of HPA Confocal Microscopy Images

**DOI:** 10.3390/bioengineering12121379

**Published:** 2025-12-18

**Authors:** Vincenzo Taormina, Domenico Tegolo, Cesare Valenti

**Affiliations:** Dipartimento di Matematica e Informatica, Università degli Studi di Palermo, 90123 Palermo, Italy; domenico.tegolo@unipa.it (D.T.); cesare.valenti@unipa.it (C.V.)

**Keywords:** transfer learning, fluorescence images, confocal microscope, images, multi-class multi-label classification, convolutional neural network, support vector machine, genetic algorithm, binary relevance, label powerset

## Abstract

Advances in deep learning are impressive in various fields and have achieved performance beyond human capabilities in tasks such as image classification, as demonstrated in competitions such as the ImageNet Large Scale Visual Recognition Challenge. Nonetheless, complex applications like medical imaging continue to present significant challenges; a prime example is the Human Protein Atlas (HPA) dataset, which is computationally challenging and complex due to the high-class imbalance with the presence of rare patterns and the need for multi-label classification. It includes 28 distinct patterns and more than 500 unique label combinations, with protein localization that can appear in different cellular regions such as the nucleus, the cytoplasm, and the nuclear membrane. Moreover, the dataset provides four distinct channels for each sample, adding to its complexity, with green representing the target protein, red indicating microtubules, blue showing the nucleus, and yellow depicting the endoplasmic reticulum. We propose a two-phase transfer learning approach based on feature-block extraction from twelve ImageNet-pretrained CNNs. In the first phase, we address single-label multiclass classification using CNNs as feature extractors combined with SVM classifiers on a subset of the HPA dataset. We demonstrate that the simple concatenation of feature blocks extracted from different CNNs improves performance. Furthermore, we apply a genetic algorithm to select the sub-optimal combination of feature blocks. In the second phase, based on the results of the previous stage, we apply two simple multi-label classification strategies and compare their performance with four classifiers. Our method integrates image-level and cell-level analysis. At the image level, we assess the discriminative contribution of individual and combined channels, showing that the green channel is the strongest individually but benefits from combinations with red and yellow. At the cellular level, we extract features from the nucleus and nuclear-membrane ring, an analysis not previously explored in the HPA literature, which proves effective for recognizing rare patterns. Combining these perspectives enhances the detection of rare classes, achieving an F1 score of 0.8 for “Rods & Rings”, outperforming existing approaches. Accurate identification of rare patterns is essential for biological and clinical applications, underscoring the significance of our contribution.

## 1. Introduction

**Figure 1:**
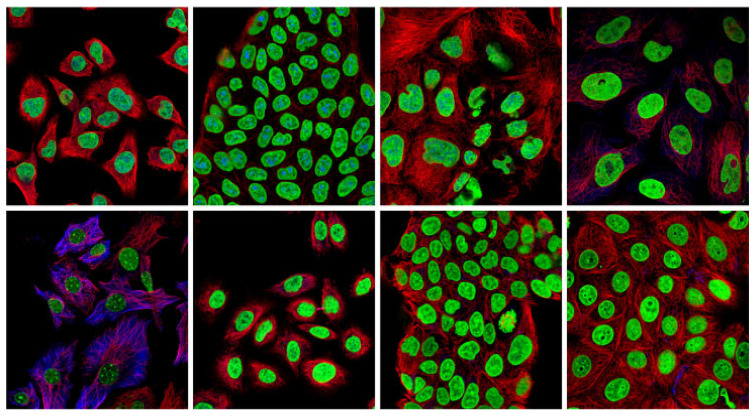
Examples of HPA samples with single-label patterns (i.e., each image shows a single protein localization pattern).

Deep learning models, particularly Convolutional Neural Networks (CNNs), have revolutionized image classification, often outperforming humans. These advances have been particularly evident in benchmark competitions such as the ImageNet Large Scale Visual Recognition Challenge (ILSVRC) [1]. However, applying deep learning in complex fields like medical imaging remains challenging, especially with highly imbalanced datasets containing rare patterns and multi-label tasks. An example of such a dataset is the Human Protein Atlas (HPA) [2], an open-access project dedicated to mapping all human proteins within cells, tissues and organs. The HPA project produced a large confocal fluorescence microscopy dataset, with over 500 unique label combinations across 28 distinct protein patterns. Figure 1 illustrates examples of HPA samples exhibiting single-label patterns, meaning that each image contains only one protein localization pattern without any mixed or multi-label combinations. Accurate classification of different protein patterns in HPA images is difficult for several reasons. First, there is a significant imbalance among classes, with some protein patterns that are quite rare. Moreover, many samples exhibit multi-class and multi-label patterns (often two, but sometimes up to six mixed patterns), complicating predictions. In addition, protein expression varies in distinct cellular regions such as the nucleus, nuclear membrane and cytoplasm. These characteristics make developing accurate and efficient models challenging. In this work, we propose a feature-based transfer learning strategy specifically designed to cope with the severe class imbalance of the HPA dataset. By combining heterogeneous architectures and complementary channel information, our approach improves discrimination of rare patterns, as shown by performance gains on classes like ‘Rods & Rings’. To emphasize the importance of this image classification task, a Kaggle competition [3] was conducted on the HPA dataset with the goal of creating automated models capable of identifying these mixed and rare patterns with expert-level precision.

Our work introduces a transfer learning approach designed to address the challenges of the HPA dataset. Leveraging well-known pre-trained CNN architectures on the large-scale ImageNet dataset [4], our approach aims for high classification performance while remaining computationally efficient. Transfer learning from pre-trained CNNs can be applied by fine-tuning or by using network layers as feature extractors with additional classifiers. In fine-tuning, the entire architecture is retrained, or some layers are frozen while the remaining layers are retrained, which can be computationally intensive. Furthermore, the presence of rare classes with very small numbers in the database makes it difficult to divide into training, validation and testing for proper generalization. Our approach takes advantage of the latter approach, in which the network layers are used as feature extractors that are fed into a simple and effective classifier. We propose a pipeline that begins with the extraction of feature blocks from layers of twelve pre-trained CNN architectures. We hypothesize that if a single feature block extracted from a known CNN has discriminatory strength for classifying the HPA dataset, then combining feature blocks from multiple CNNs with different architectures should further enhance the discriminatory ability.

To manage computational resources efficiently, we employ a two-stage strategy. In the first phase, we tackle a multi-class, single-label classification task using a subset of the HPA dataset. We use layers of CNNs as feature extractors by processing features using Support Vector Machines (SVMs) with linear kernels. Our experiments reveal that the concatenation of feature blocks from different CNNs can improve classification performance and we refine further this approach by applying a Genetic Algorithm (GA) to identify the sub-optimal combination of feature blocks. GA was chosen for its efficiency in searching large combinatorial spaces, where exhaustive methods are infeasible. In our work, we carried out a search over 2^72^ possible feature block combinations at the image level, making GA a computationally efficient and suitable choice. In the second stage, based on the results of the initial phase, we apply two common multi-label classification strategies: Binary Relevance (BR) and Label Powerset (LP) and compare their performance with four standard classifiers. Our methodology integrates image- and cell-level perspectives and presents the results of each level individually as well as in combination. At the image-level, considering that each sample comprises four distinct image channels, we examine which channels or combinations of channels offer the highest discriminatory ability. This is based on the fact that the HPA dataset consists of four distinct fluorescence channels for each sample, each providing different information: the green channel shows the target protein stained by a specific antibody, the red channel highlights microtubules, the blue channel highlights the cell nucleus, and the yellow channel represents the endoplasmic reticulum. At the cellular level, we analyze the features of the nucleus and nuclear membrane ring, whose joint analysis has not been previously investigated in the HPA literature. This dual cellular-region formulation provides complementary structural information that proves particularly beneficial for the detection of rare patterns. To the best of our knowledge, this is also the first study to systematically explore feature-block extraction from twelve pre-trained CNN architectures, combining them through Genetic Algorithm optimization to navigate an inherently large combinatorial space (2^72^ possible feature-block configurations). This design allows us to exploit the diversity of architectural representations together with the complementary information provided by the four imaging channels.

We evaluate our approach using measures such as the F1 macro score, as in the original HPA competition, to assess classification quality, particularly for rare classes. Our results show an F1 macro score of 0.59, a weighted F1 score of 0.68, a precision of 0.71, and a recall of 0.66, thus demonstrating competitive performance without the need for extensive retraining or fine-tuning. Notably, our method achieves F1 scores of 0.8 for “Rods & Rings” and 0.4 for “Microtubule Ends”, thus outperforming most existing approaches in the literature for these challenging rare patterns.

To our knowledge, this is the first application of a feature-based transfer learning strategy to the HPA problem, achieving competitive or superior results compared with studies using new or fine-tuned deep models, and the first application of a cell-level approach that processes the nucleus and surrounding nuclear membrane region.

The main contributions of this study are as follows:

- A dual-level classification framework integrating both image-level and cell-level analysis, introducing two regions of interest at the cellular level (nucleus and nuclear membrane), whose joint use has not been previously investigated in the HPA literature.
- Feature-based transfer learning across twelve pre-trained CNN architectures, one of the most comprehensive evaluations applied to the HPA dataset.
- GA for selecting sub-optimal combinations of CNN feature blocks, addressing the high-dimensional combinatorial search space (2^72^ combinations).
- Investigation of discriminative channel combinations in multi-channel fluorescence microscopy.
- Two-phase, computationally efficient strategy for multi-class and multi-label tasks, avoiding costly fine-tuning while achieving strong performance, especially for rare classes.

The rest of this paper is organized as follows: Section 2 discusses related works, whereas Section 3 provides an overview of the materials and methods, including the HPA dataset, the CNNs utilized, the proposed two-phase strategy with preprocessing steps, feature block extraction and classification. Section 4 presents the experimental results and Section 5 concludes by summarizing our findings and discussing potential directions for future work. Additional data are given in the Appendix A.

## 2. Related Work

Despite the variety of approaches applied to the HPA dataset, many existing methods face notable limitations. Several models restrict their analysis to a subset of classes or images, thereby reducing the complexity of the classification task and limiting generalization. The authors of the HPA database and competition [2] summarized the approaches used by participants, noting that the winning model achieved a F1 macro score of 0.59. However, this result remains far from the estimated value for human experts, which is 0.71, as indicated by the authors. The team that achieved the best performance used an optimized DenseNet architecture, believing it to be more effective than ResNet. The GlobalMaxPool and GlobalAvgPool layers of the final CNN feature map were concatenated before being fed to two fully connected layers to calculate the probability of each class. Multi-Label Stratification was used to split the dataset into a training set and a validation set. Data augmentation included flipping, rotating and random cropping. During testing, multiple crops were considered, and the maximum expected probability was used. In [2], the analysis of the numerous contributions highlights the importance of assembling different smaller model pipelines, trained using both the whole image and segmented cells for subcellular localization of proteins in the HPA dataset.

However, we observed that the various contributions to the competition were not subsequently published by the respective authors in international scientific journals. We identified [5] where this study introduces a composite Focal-Lovász loss function. Using CNNs (ResNet-50, DenseNet-121 and SE-ResNeXt-50) the authors demonstrate that Focal-Lovász loss outperforms traditional loss functions by effectively addressing minority classes. That study applies data augmentation techniques, including flips and rotations, and uses all four image channels as inputs to the pre-trained CNNs. DenseNet-121 achieves the best individual performance among the three networks through 5-fold cross-validation. The use of a model ensemble technique yields a F1 macro score of 0.529. GapNet-PL model, a CNN-based approach for multi-label classification on the HPA dataset, is introduced in [6]. To resolve data imbalance, a combination of oversampling and undersampling techniques was employed, along with data augmentation through geometric transformations. The dataset was divided using five-fold cross-validation. After training all five models, their results were averaged to obtain the final output. That study evaluated the effectiveness of Parametric Rectified Linear Unit (PreLU) and Scaled Exponential Linear Unit (SeLU) activation functions, finding that GapNet-PL with PReLU achieved an average F1 score of 0.541. Combined citizen science and deep learning to improve HPA classification is presented in [7]. A mini game called Project Discovery was integrated into the video game EVE Online, involving 322,006 players who contributed nearly 33 million subcellular localization annotations, including previously unannotated patterns. These annotations were combined with the deep learning model Loc-CAT, which classifies proteins into 29 localization patterns and handles multiple localization proteins in different cell morphologies. Taking advantage of transfer learning, that approach achieved an F1 macro score of 0.47.

In some works, proposed methods have been based on different subsets of the HPA dataset, varying in the number of classes selected, the total images used and the degree of class imbalance considered in their evaluations. The authors of [8] propose MMLoc, a deep learning-based model formulated as a multi-instance multi-label learning task. Using the HPA dataset, they focused on eight patterns with a total of 5772 images. Each image was segmented with a U-Net to produce 1 to 90 cell patches, with an average of 11.6 patches per image. These patches were grouped into 13,532 instance bags with 12 cells per bag. Features were extracted using pre-trained ResNet-50 and DenseNet-121 models. The method achieved an F1 score of 0.819. In [9], protein localization was explored using the HPA dataset, focusing on only 13 classes and using 20,000 images. Two ad hoc deep learning architectures were proposed: a CNN and a Fully Convolutional Network (FCN). Both networks processed all four channels of each sample, employing a simple yet effective design with 10 convolutional layers. Those results showed that FCN slightly outperformed CNN with an average percentage of correctly classified samples of 0.676 for CNN and 0.696 for FCN. 14,094 samples from 15 different classes of the HPA dataset were used to compare two classification approaches: a conventional method relying on handcrafted feature extraction and a CNN-based method using ResNet50 and Xception architectures [10]. The conventional approach extracted Haralick features, Local Binary Patterns, Zernike moments and channel comparisons, which were classified using a Random Forest classifier. In contrast, the CNN-based approach employed deep learning models directly on the image data. The results showed that the Hybrid Xception model achieved the best performance with an average F1-score of 0.69, significantly outperforming the conventional approach, which achieved an F1-score of 0.61. SIFLoc, a two-stage method combining self-supervised pre-training and supervised learning to improve classification from HPA images, was introduced in [11]. Using a dataset of 19,777 images organized into 1600 initial bags and further split into 13,261 smaller bags containing 173,594 patches, the method leverages RGB images constructed from the red, green and blue channels. Experiments tested three train:val:test ratios (2:1:7, 4:1:5 and 6:1:3) achieving F1 macro scores of 0.353, 0.403 and 0.399, respectively.

Among the studies that have explored the use of transfer learning approaches from pre-trained networks is [12]. The authors proposed a method using three well-known pre-trained CNN architectures: VGG16, ResNet152 and DenseNet169. Their approach involved fine-tuning these networks to fit the specific characteristics of the HPA dataset, followed by the application of a stacked ensemble technique based on the neural network. They used three out of the four available channels, specifically combining the red, blue and green channels to create a three-channel composite image as input for the CNN models. They split the data into 80% and 20% for training and testing, respectively, using an iterative stratification method. Their results demonstrated that the stacked ensemble technique outperformed the individual networks, achieving a weighted F1-score of 0.71. The authors of [13] tackle the data imbalance issue by proposing an oversampling method that generates synthetic samples using a combination of data augmentation and an imbalance-aware sampling approach. The score of concurrence among imbalanced labels measures is used to compute the imbalance ratio per class and the agreement between majority and minority classes. Synthetic samples are created through nonlinear mix-up, geometric transformations and color transformations in the 3D color space obtained by the red, green and yellow channels. That method was evaluated on the HPA dataset by randomly selecting 20% of the images in each class as a test set and the remaining data was divided into training (80%) and validation (20%) sets for a 5-fold cross-validation. ResNet-50 was tuned for classification and obtained an F1 macro score equal to 0.478.

Overall, the reviewed literature demonstrates notable progress, yet most methods still fall short of expert-level performance (macro F1 ≈ 0.71). A critical limitation concerns the accurate classification of rare localization patterns. In contrast, our method exhibits improved robustness in these underrepresented classes.

To better illustrate the methodological differences and, crucially, the specific scope of data analyzed across these studies, we summarize the key contributions in Table 1, highlighting their dataset limitations and main technical focuses.

## 3. Materials and Methods

This section details the materials, preprocessing steps and the methodologies used to address the challenges of highly unbalanced multi-label classification within the HPA dataset. We extend our prior work [14], in which we focused on single-label classification using a subset of the dataset.

### 3.1. Human Protein Atlas Dataset

**Figure 2:**
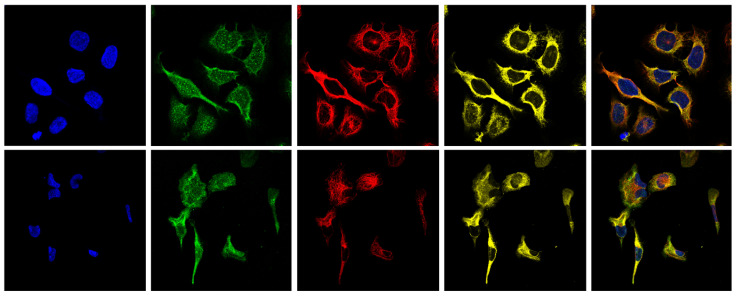
Each row presents a distinct sample from the HPA dataset, displayed across its four fluorescence microscopy channels: the blue channel delineates the nucleus, the green channel visualizes the antibody-stained target protein, the red channel highlights the microtubule network, and the yellow channel maps the endoplasmic reticulum. The rightmost panel in each row shows the composite image obtained by merging the four channels, providing an integrated representation of the subcellular structures.

The Human Protein Atlas dataset [2], part of an open access initiative, is dedicated to creating a publicly available atlas of all human proteins in cells, tissues and organs. This dataset contains an extensive annotated collection of fluorescence microscopy images with over 42,000 samples of human cells acquired by confocal microscopy. It presents a challenging classification problem due to its highly imbalanced, multi-class nature, featuring 28 distinct protein patterns that appear individually or in various combinations, resulting in more than 500 unique combinations of labels. Each sample in the HPA dataset consists of four fluorescence microscopy images representing different cellular components: the target protein, highlighted in the green channel by a specific antibody; microtubules in the red channel; the cell nucleus in the blue channel; and the endoplasmic reticulum in the yellow channel. Figure 2 illustrates, for each of the two representative samples, the four individual fluorescence channels together with the corresponding composite image, whereas Table A1 in the Appendix A provides the complete list of 28 protein patterns.

The HPA dataset is divided into a public training set of 31,072 samples and a private test set of 11,702 samples. Overall, the dataset includes more than 171,000 images available at the original high resolutions (i.e., 2048 × 2048 or 3072 × 3072 pixels) in uncompressed TIF format, totaling approximately 250 GiB. In addition, a downscaled version (i.e., 512 × 512 pixels) is available in lossless PNG format, with a total size of roughly 19 GiB. All images are saved in 8 bits grayscale format. This dataset is highly unbalanced, with rare patterns such as “Rods & Rings” and “Microtubule Ends”.

### 3.2. Pre-Trained CNN

We use the feature extraction capabilities of twelve well-known CNN architectures, pre-trained on the renowned ImageNet dataset of the ILSVRC challenge [1]. Their success is due to their ability to find discriminative features during training across multiple layers of convolutional kernels by directly exploiting images without explicit feature engineering. However, training these networks from scratch requires substantial computational and power resources. Thanks to transfer learning methods, these pre-trained CNNs offer a significant environmental advantage in that they allow existing models to be reused on other datasets [15]; this reduces computational costs and power consumption associated with training from scratch.

There are two main approaches to transfer learning for using pre-trained CNNs in other classification tasks. The first is fine-tuning, in which the entire architecture is retrained, or some layers are frozen while the remaining layers are retrained on new data. Although fine-tuning can adapt well to new tasks, it can be computationally intensive depending on the number of layers and parameters to be retrained. The second approach uses the network layers as feature extractors, which are fed into a possibly simpler and more computationally efficient classifier. The choice of classifier and its parametrization can further reduce the computational requirements in this transfer learning approach. While fine-tuning may require a significant amount of training data to generalize well and avoid overfitting on new tasks, the use of pre-trained CNNs as feature extractors is less data-dependent and can generalize well with smaller datasets. This is particularly relevant to the HPA dataset, where some rare classes have very few samples, making it difficult to partition the data for effective training, validation and testing. To effectively manage computational resources and to avoid overfitting on these rare classes, we use the CNNs as feature extractors, exploiting their pre-learned representations. This approach efficiently captures both rich and general image features, which can then be classified by an efficient and effective classifier that offers robust performance with reduced training requirements. Given the peculiarities of different CNN architectures, we selected twelve widely used pre-trained CNNs as feature extractors, all trained on ImageNet (see Table 2).

AlexNet [16], winner of ILSVRC 2012, was the first deep CNN to showcase the potential of deep learning for large-scale image recognition. Its architecture includes five convolutional and three fully connected layers, uses ReLU activations for faster training, and applies dropout in the fully connected layers to reduce overfitting. It set a new standard and inspired a generation of deeper models. VGG-16 and VGG-19 [18], introduced in ILSVRC 2014, adopt a straightforward architecture with stacked 3 × 3 convolutions and max-pooling. Their greater depth enables deep feature hierarchies, though at the cost of more parameters and computational demand. MobileNet-v2 [21] is optimized for mobile and embedded systems. It uses depthwise separable convolutions and inverted residuals with linear bottlenecks, achieving a good trade-off between accuracy and efficiency. SqueezeNet [22] targets parameter efficiency. It replaces most 3 × 3 filters with 1 × 1 convolutions and introduces fire modules, achieving AlexNet-level performance with a 50× smaller model size, ideal for memory-constrained applications. ResNet-18, ResNet-50 and ResNet-101 [19] belong to the residual network family that won ILSVRC 2015. Their key innovation is the use of shortcut connections forming residual blocks, which makes it easier to train deeper networks by avoiding vanishing gradients. GoogLeNet [17], Inception-v3 [20], and InceptionResNet-v2 [24] follow the Inception architecture, which performs multi-scale processing using parallel convolutional paths. GoogLeNet, winner of ILSVRC 2014, reduced parameters through 1 × 1 convolutions. Inception-v3 improved this with factorized convolutions, while InceptionResNet-v2 added residual connections to support even greater depth. DenseNet-201 [23] uses dense connectivity, where each layer receives inputs from all previous layers. This encourages feature reuse, reduces redundancy, and improves gradient flow, making it efficient despite its high depth.

The twelve CNN architectures chosen for this study bring distinct strengths based on their diverse structures and depths. Using a different set of pre-trained CNNs, we capture a broad spectrum of rich and generic features, supporting a robust transfer learning strategy. We also analyze how combining features extracted from different CNNs can improve performance, also considering their diversity.

### 3.3. Proposed Two-Phase Sustainable Method and Data Splitting

To reduce the computational load in terms of time and hardware resources, we adopted an incremental update approach divided into two phases. In the first phase, the subset of the HPA dataset included only single-class samples from the public training set, excluding multi-labeled images. This decision was made to focus on identifying features and patterns that could discriminate effectively among different classes in the dataset. By discarding multi-labeled cases from the training set, we reduced the dataset to about 15,000 samples out of the total 31,000 (i.e., practically 50% of the data). Though the dataset contains 28 distinct classes, we accessed only 24 classes in this first phase because the classes “Lysosomes”, “Microtubule End” and “Mitotic Spindle Microtubule” had no single-class samples, whereas the class “Rods & Rings” had just one sample. The goal of this first phase was to identify quickly discriminating feature blocks and promising pattern configurations. We used 4494 samples as the training set; the remaining 10,631 samples in the dataset were used as the test set. Given the highly imbalanced data, we tried to balance, where possible, the various classes in the training set, leaving the excess samples in the test set. The selection of samples to be included in the training set was done randomly and we set a maximum threshold of 250 samples for sufficiently large classes. For classes with fewer than 275 samples, we allocated about 80% of the samples to training and 20% to testing. This approach balanced the training set while keeping the test set highly unbalanced. In the second phase, the whole HPA dataset was used for multi-class multi-label classification. We took advantage of the knowledge gained in the previous stage and applied it to the entire multi-label dataset with a two-sample multi-label strategy combining specialized single-class classification models at both the image level and the cell level. We applied the iterative stratification strategy [25] with an 80%–20% split between training and testing as in [12]. In particular, we made sure that the 4494 training samples from the first phase remained in the training set of the second phase to avoid eventual bias.

### 3.4. Image Preprocessing and Segmentation

To extract features from the CNN layers, we preprocessed the HPA samples to feed the twelve pre-trained CNNs with appropriate images. Indeed, these CNNs were designed to receive input images with three channels and 24 bits per pixel (i.e., 8 bits per pixel in each channel). Furthermore, these input images must have precise spatial resolution and we report the input dimensions specified in Table 3. As mentioned above, we worked both at the image level and at the cellular level. In the former case, resizing to the dimensions of the input CNN image is performed by bicubic interpolation using the scaled version of the HPA samples. Considering that a sample consists of four channels, in cases where only one channel was used to extract features, we replicated that channel three times (i.e., we obtained a grayscale image). As suggested in [12], we created composite images using the blue, green, and red channels to study the influence of different channels; moreover, we considered the average of all four channels, replicating the average for three channels (i.e., we again obtained a grayscale image).

**Figure 3:**
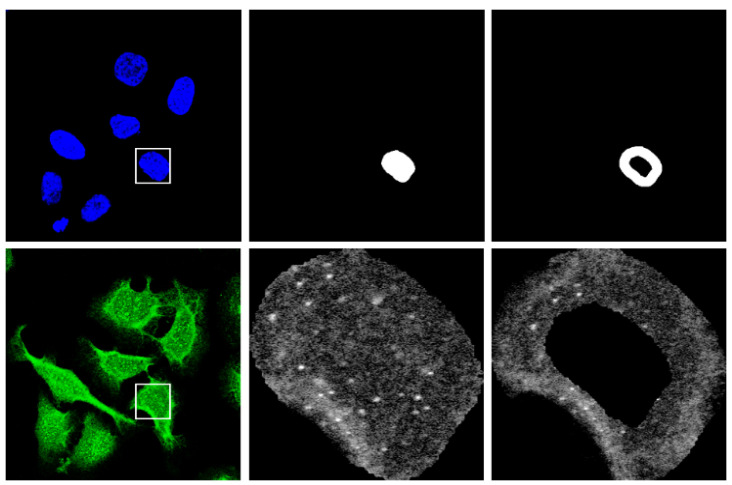
Example of the segmentation process (detail within the square). Top row: (**Left**) Blue channel scaled image (512 × 512) used for segmentation. (**Center**) Binary mask of a segmented nucleus. (**Right**) Binary mask of the segmented ring. Bottom row: (**Left**) Original full-size image of the green channel. (**Center**) Segmented nucleus. (**Right**) Segmented ring around the nuclear membrane.

When working at the cellular level, accurate segmentation of cells is crucial. We leverage existing and well-established methods suggested in [3]. Specifically, we use the Cellpose library [26] through the Medical Imaging Toolbox of MatLab2024a [27]. Cellpose offers a suite of pre-trained models optimized for cell segmentation in microscopy images. We opted for the “nuclei” model because it excels in scenarios where nuclei are clearly demarcated, as in the case of our blue channel. We used the scaled version of the blue channel (i.e., 512 × 512 from the PNG file) because this channel served as a reference for nuclei segmentation [2]. We used the default parameters of Cellpose with the “nuclei” model, setting the average diameter of the nucleus to 55 pixels. Notably, using scaled images instead of full-sized ones significantly improved the processing speed, reducing the computation time from approximately 60 s to just 1 s per image on our system (see Section 4) without a substantial difference in the quality of the segmentation mask. It is important to note that the scaled images from the HPA dataset appear more pixelated than the full-size images. For this reason, we obtain the segmentation mask by working on the blue channel of the resized sample, but apply the binary mask to the green channel of the original full-size sample to segment the cells. To achieve this, we simply resize the binary mask (i.e., 512 × 512) to match the size of the original images (i.e., 2048 × 2048 or 3072 × 3072). We removed all segmented nuclei located at the borders of the image and those with an area less than a minimum threshold of 100 pixels. For each nucleus, its bounding box is cropped, zeroing out background pixels outside the nucleus area. In addition, because some patterns present relevant information about the nuclear membrane, an annular region surrounding this membrane is extracted. This is done by applying a morphological dilation to the nucleus mask and then subtracting an eroded version of the original mask. For both dilation and erosion, a 5 × 5 disk-shaped structuring element is used. Figure 3 illustrates an example of a segmented nucleus and a surrounding ring that incorporates the relevant nuclear membrane region. No additional preprocessing steps are applied to the images other than resizing for input to the CNNs (refer to Table 3 for specific sizes accepted by each CNN).

### 3.5. Features Block Extraction and Selection with Genetic Algorithm

Feature extraction was performed at both the image and cell levels. At the cellular level, we extracted specific features from individual nuclei and the surrounding area, incorporating the nuclear membrane. We refer to these as feature block extractions because we used the layers of pre-trained CNNs on ImageNet as rich and generic feature extractors. For this study, we favored using a set of well-known CNNs rather than exploring various individual layers. We selected the last layers of each CNN as shown in Table 4. Through feature extraction using these layers, we obtained feature blocks, each consisting of 1000 features corresponding to the number of object classes that CNNs have been trained to recognize in ImageNet.

Although the main channel for pattern detection is the green channel [2], at the image level, we explored the influence of the other channels as well. Therefore, feature extraction was performed separately on different channels: the four available channels (blue, green, red and yellow), the composition of the blue-green-red channels [12] and the average of all four channels, resulting in six different feature extractions per sample. At the cellular level, feature extraction was performed on the green channel for both the nucleus and the surrounding nuclear membrane. This choice was motivated by the results obtained at the image level and the fact that the red and yellow channels provide relevant information about cytoplasm, whereas the blue channel mainly highlights the nucleus. The first step of our method is designed to identify even the most relevant features. Our goal is to select the most suitable feature blocks to solve the classification problem at both the image and cell levels. Considering the extraction of feature blocks using a single channel (e.g., the green channel), we have up to 12 feature blocks available, each comprising 1000 distinct features. It is possible to perform ensemble methods by simply concatenating some or all of these blocks.

As the results show, the ensemble of multiple feature blocks outperforms individual feature blocks. This corresponds to our hypothesis: if a block of 1000 features extracted from a pre-trained CNN has a certain discriminatory ability in classifying the HPA dataset, then the combination of two or more blocks extracted from CNNs with different architectures will exhibit higher discriminatory ability in classification. If we were to conduct an exhaustive search on the 12 blocks to identify the combination that optimizes classification, we would have to test 2^12^ = 4096 different combinations of blocks. Furthermore, if we explore the influence of other channels (as we did at the image level), the number of combinations increases significantly, making this approach impractical. At the image level, we proposed six different channel types from which to extract the 12 feature blocks, for a total of 12 × 6 = 72 extractable feature blocks per sample; in this case, their combinations would be 2^72^ = 4,722,366,482,869,645,213,696.

To approach the selection of the most effective blocks from a non-exhaustive perspective while obtaining good sub-optimal solutions, we leveraged the power of Genetic Algorithms [28]. GA is a global optimization technique with an underlying random approach to search for solutions in a large search space using evolution-inspired methods. From now on, we will use the term random to indicate a pseudorandom value according to a uniform probability distribution; this leads to a powerful and suitable tool for selecting an optimal subset of feature blocks in a probabilistic sense. We want to show that the results obtained by GA outperform the ensemble of all feature blocks extracted. Possible solutions, so-called individuals, improve from generation to generation toward better solutions by mating each other to pass on a better genetic endowment to their offspring. Occasionally, this genetic information is subject to mutation to ensure the evaluation of otherwise unreachable areas of the search space. In our case, an individual is represented by a binary vector whose length corresponds to the number of feature blocks extracted. A value equal to 1 in the vector indicates that that feature block is included; vice versa, 0 indicates its absence. Essentially, if the vector consists entirely of 1, then all feature blocks are considered; conversely, if it consists entirely of 0, none are considered. At the image level, each individual is represented by a binary vector of 72 values. At the cellular level, each individual is represented by a binary vector of 24 values, derived from 12 feature extraction layers across two distinct cellular regions of interest: the nucleus and the surrounding nuclear membrane.

A fitness function applies natural selection because it evaluates how close a given individual comes to the solution. In general, the search process ends after a predetermined number of generations or when an acceptable solution is found. We defined the fitness function as the F1 macro score classification performance achieved during training and testing with the feature blocks selected by the individual. For performance reasons, we set the population size at just 16 individuals across 25 generations. At the beginning of each new generation, these 16 individuals are randomly permuted to form 8 random mating pairs. For each of these 8 pairs, we apply single-point crossover. The crossover point is also chosen randomly with the constraint that it cannot be at the initial and final positions (to avoid full swapping of individuals). For mutation, a fixed mutation rate of 0.125 is used, meaning that at each generation, mutation is applied to 2 individuals out of 16. Mutation is performed by randomly selecting a position in the binary vector and negating its value. After applying both crossover and mutation, we verify whether the new individuals represent solutions already considered: if a duplicate solution is found, we apply the mutation operator again until a previously unexplored solution is generated. Parents are evaluated together based on their fitness values: only the top two individuals among each group of four (i.e., two parents and their two offspring) are retained for the next generation. This localized selection strategy helps maintain population diversity and reduces the risk of premature convergence to local optima, compared to globally selecting the best individuals across the entire population.

### 3.6. Classification Strategies: Multi-Class and Multi-Label Approaches

To deal with the multi-class single-label classification task in the first step, we selected the effective and widely used SVM classifier, proposed in the 1990s [29]. SVM is classical in supervised machine learning and has been applied extensively for both classification and regression tasks [30]. We opted for this classifier for its computational efficiency and ease of parametrization while maintaining high performance. Given the high dimensionality of the feature vector in the first phase, where the concatenation of various feature blocks exceeded 20,000 dimensions in some cases, we chose a linear kernel for the SVM, which is fast and simple in terms of parameter tuning, as it mainly requires adjusting only the penalty parameter “C”. A standard approach to addressing a multi-class problem is to divide an N-class instance into a series of binary, or two-class, problems [31,32]. The two main methods employed are the One-Against-All (OAA) and One-Against-One (OAO) techniques. OAA divides a dataset of N classes into N separate instances of two classes. In this method, each of the N classifiers is responsible for distinguishing one class from all others, which results in 24 SVMs in our specific case. For a new sample, a class is assigned by evaluating the results of each classifier and typically selecting the class with the highest score. However, this approach assumes that all classifiers involved in the decision are equally reliable, which is seldom true in practice. In contrast, the OAO method constructs one classifier for each possible class pair, resulting in a total of N(N − 1)/2 classifiers (i.e., 276 SVMs in our case). Although OAO may be more computationally demanding, it assigns a label to a sample by assuming the votes of each binary classifier and the class receiving the most votes is selected as the final label. In a highly imbalanced multi-class problem with diverse patterns, the large number of binary classifiers may allow each classifier to focus more effectively on the specific distinctions between two patterns, potentially mitigating the imbalance issue. However, a limitation of OAO is that it aggregates votes without considering the relative reliability of individual classifiers when they are asked to rate patterns other than those for which they were trained, which can lead to inconsistencies in classification performance. Given these considerations, while in the first stage we compared both strategies, in the second stage, we exploited the results of all 276 binary classifiers produced by the OAO strategy as new discriminant features. This approach not only overcomes the voting limitation but also allows a significant reduction in the dimensionality of generic features extracted from the CNN layers. Moreover, these second-level features can be interpreted as a refined feature set, specifically tailored to distinguish among the complex patterns of the dataset. This method aligns with a stacking strategy, in which the outputs of multiple base classifiers (in this case, the binary SVMs of the OAO strategy) are used as input to a metaclassifier rather than relying on a simple voting mechanism. By integrating features derived from the OAO strategy, we build a more robust, specialized and compact set of second-level features. This refined set captures intricate pattern distinctions, enabling more effective resolution of the complex multi-class multi-label classification problem. Through this approach, we exploit a reduced set of specialized features, suitable for various models within the HPA dataset, while balancing computational cost and performance.

To integrate image-level and cell-level information into the final multi-label classification, we adopted a simple concatenation strategy. For each image, we computed 276 second-level features at the image level, derived from the outputs of the OAO SVM models trained in phase 1 for image-level classification. We also computed 276 second-level features at the cellular level, leveraging the OAO SVM models trained in phase 1 for cell-level classification. The latter were obtained by averaging the outputs of the 276 binary SVMs across all valid segmented cells in the image, thereby standardizing the cellular information into a fixed-length (276-dimensional) vector for every image. These two vectors were then concatenated, resulting in a unified 552-dimensional feature vector that captures both global and local discriminative patterns. This combined representation was used as input for the multi-label classification strategies.

To address the multi-label classification problem using our second-level feature set, we apply two widely used multi-label classification strategies: Binary Relevance (BR) and Label Powerset (LP) [33]. Although they are conceptually similar to OAA and OAO strategies, these are intended for multi-class problems. BR transforms a multi-label classification problem into multiple binary classification tasks by creating a classifier for each label independently. This simpler approach is flexible, since each classifier makes separate predictions for each label. LP converts a multi-label classification problem into a single multi-class task by treating each unique combination of labels as a distinct class. This method effectively captures label dependencies since it treats each combination as a unique pattern. However, LP can become computationally demanding when there are numerous label combinations. Despite the higher complexity of LP, we can apply it to the HPA dataset due to the low dimensionality of the second-level features and the choice of simple and efficient classifiers. In this multi-label phase, in addition to using SVM with a linear kernel, we also use three other simple classifiers, such as Logistic Regression [34], Decision Tree [35] and K-Nearest Neighbors (KNN) [36] to provide a comparative analysis. In the first phase, we used a cross-validation strategy to build the SVM models and optimize the penalty parameter “C”, specifically employing 5-fold cross-validation. The training data, consisting of 4494 samples, was divided into five segments, where iteratively one segment served as validation and the remaining segments were used for training. The model’s performance was averaged over five folds to obtain a more reliable performance. The optimization focused on maximizing the F1 macro figure of merit [37], a measure that gives equal weight to all labels, which is particularly advantageous in unbalanced problems where some labels are less frequent. During each cross-validation fold, feature vectors were normalized using the mean and standard deviation computed exclusively from the training portion of that fold. These statistics were then applied to normalize the corresponding validation set, preventing data leakage. After cross-validation, we built the final SVM models using the selected parameters on all 4494 training samples. These models were then evaluated on the test set, comprising 10,631 samples. To this end, the entire training set was normalized using its own mean and standard deviation (Z-score standardization), and these same statistics were subsequently used to normalize the test set to prevent data leakage during classification.

In the second phase, we applied an 80–20% training-test split using the iterative stratification strategy [25], which considers all combinations of labels, maintaining proportions among sets and preventing rare labels from appearing exclusively in the training set or the test set. This method calculates label frequencies and iteratively assigns instances accordingly. Since the outputs of the first-level SVM classifiers are probability estimates, which are already normalized, no additional normalization was performed on the second-level feature set.

**Figure 4:**
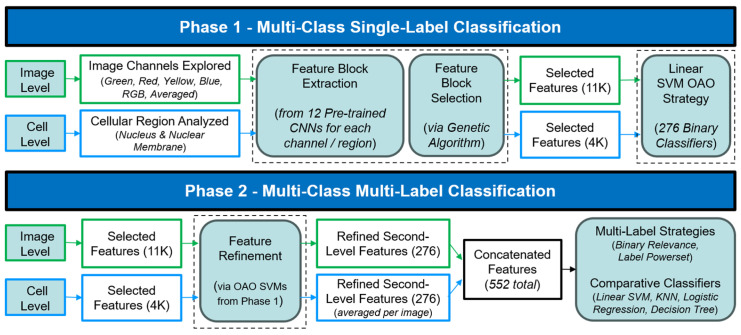
Workflow of the Proposed Transfer Learning Approach for HPA Sample Classification. The method comprises two main phases: Phase 1 focuses on multi-class single-label classification using a subset of the dataset to identify sub-optimal feature blocks concatenations (from various channels/cellular regions) via Genetic Algorithm, and train binary SVM classifiers (OAO strategy). Phase 2 leverages these trained models to generate refined, second-level features from the full HPA dataset for multi-label classification using various strategies and classifiers. Blocks enclosed by a dashed line represent common procedures applied at both image and cell levels, albeit with distinct inputs and outputs.

A comprehensive overview of the entire workflow, from data input to final classification, is provided in Figure 4.

## 4. Experimental Results

The experimental results are presented in relation to the two phases already described. For all experiments, we used an HP Elitebook with 16 GiB RAM, an Intel Core i7 8th Gen processor and Windows 10 to demonstrate that our approach does not require high-performance hardware. MatLab2024a [27] was used for image processing, segmentation and feature extraction, whereas the free version of Google Colaboratory (COLAB) (2025-11-13) [38] was employed for SVM model construction and classification. The experimental results are presented in relation to the two phases already described. Section 4.1 presents results for the multi-class single-label task at image and cell levels, while Section 4.2 addresses the multi-class multi-label problem, including stacking-based second-level features and integration of both levels. In addition to reporting quantitative performance and related discussion, both subsections also detail the computation times required at each stage of the proposed pipeline, thereby providing a clear overview of the practical computational effort involved.

### 4.1. Multi-Class Single-Label Results

In the initial phase, we focused on multi-class single-label classification, analyzing data at both the image and cell levels. At the image level, we experimented with six different channel composition methods, while at the cell level, two distinct regions from the green channel were analyzed: the nucleus and the surrounding area that includes the nuclear membrane. At the cell level, segmentation generates multiple regions of interest per image. However, considering the goal of the first phase, which was to identify good solutions for tackling the subsequent multi-label problem, we reduced the computational load by selecting only one region of interest per image. We simply chose one representative cell per image based on the median area among the segmented cells. This approach ensured that the number of images processed for feature extraction was identical at both the image and cell levels. Feature extraction was performed on the public portion of the HPA database with a single label, comprising approximately 15,000 single-label samples. For this subset, the average feature extraction time for each of the six channel combinations and the two distinct cellular regions was about 631 min, which is an average of 43 min per CNN. As expected, the extraction time varied across CNN architectures because of their unique characteristics, such as differing depths and computational complexities. Table A2, in the Appendix A, summarizes the average feature extraction times for each CNN. AlexNet, being the least deep network, had the shortest extraction time, while InceptionResNet-v2, the deepest, had the longest. We emphasize that the average extraction time not only depends on the depth but also on the type of network; for example, the two VGG networks, while being among the shallowest, have one of the highest extraction times. The average extraction time for a single image ranged from approximately 0.03 to 0.37 s on our standard computer. These results confirm that computational cost is strongly influenced not only by network depth but also by architectural design, indicating that deeper models are not necessarily more expensive if equipped with efficient building blocks such as residual or inception modules. After feature extraction, we trained the SVM models using a linear kernel in both OAA and OAO configurations, obtaining 24 and 276 binary models, respectively. The regularization parameter “C” was optimized using 5-fold cross-validation on the values {0.01, 0.1, 1, 10}. Training and testing were performed using Python 3.14.2 on Google Colab, leveraging the sklearn library [39]. The default settings of the sklearn library were used for all other SVM parameters. In the first phase, at the image level, we evaluated the performance of each feature block extracted from the 12 CNNs individually, repeating this process for the six channel combinations selected in our study. The training time for the OAO configuration averaged around 4 min, while OAA took about 12 min. The OAO configuration, besides being faster, produced more competitive results on average, considering the F1 macro figure of merit. This consistent gap between OAO and OAA highlights that the intrinsic class imbalance of the dataset heavily penalizes OAA, whose one-vs-all formulation tends to generate highly heterogeneous negative classes, ultimately leading to less stable decision boundaries. The best image-level performance was obtained with the green channel and DenseNet-201 as the feature extractor.

We investigated the effect of concatenating the features extracted from the twelve layers to test the hypothesis that combining these characteristics could produce better performance. A straightforward strategy was applied, in which the features from the layer with the best result were concatenated with those from the layer with the second-best performance. Next, we added features from the third-best layer, and so on. Finally, we concatenated the features from all twelve layers, resulting in a total of 12,000 concatenated features. As shown in Table 5, which focuses on the green channel, combining features from multiple layers improves performance.

Notably, the performance obtained by concatenating features from all twelve layers is lower than that achieved by concatenating only selected layers. The decline observed when concatenating all twelve layers indicates that simply increasing feature dimensionality does not guarantee better performance and may introduce redundancy that hampers generalization. The green channel provides the highest discriminatory power, while the three channels—red, yellow and blue—contribute significantly less. The marginal contribution of the red, yellow and blue channels suggests that the most discriminative signal is encoded in protein localization (green channel), while structural channels contribute only weakly at this granularity. This observation anticipates the need for feature-selection mechanisms able to down-weight non-informative channels. However, the combined RGB channel and the average of the four channels show slightly better performance than the individual channels. Considering this minimal contribution, we evaluated whether a combination of features extracted from the green channel and selected features from the other channels could improve the overall classification performance. To further optimize performance, we applied a GA to identify the suboptimal combination of features from different layers that would maximize classification performance. This approach seeks to find the subset of feature blocks from various layers that contribute most effectively to the overall model performance. Given the diverse potential of the six channels analyzed, the initial GA population was designed to leverage the stronger potential of the green channel and the concatenation of the best-performing layers. Considering that the OAO configuration consistently outperformed OAA in both performance and computational efficiency, each individual in the GA was evaluated by the OAO method to determine its fitness. The GA evolved for 25 generations, resulting in a total of 400 evaluations. The individual with the highest fitness comprises 11K features: 8K derived from the green channel, which confirms its role as the most representative channel for discriminating various patterns, and 3K from the RGB, red and yellow channels, which contribute minimally to discrimination as hypothesized. The blue channel, as expected, was not selected, aligning with its primary importance for cell nuclei segmentation rather than classification. To test whether feature selection across all channels outperformed feature blocks extracted only from the green channel, we ran a GA restricted to the 12 blocks of the green channel. The best performing individual in this configuration included the same 8K features derived from the green channel as those selected in the best individual described above.

Table 6 summarizes the progression of the results obtained at the image level. The best performing individual achieved an F1 macro score of 36.81%, representing a gain of nearly 9% over the best result obtained using a single layer. Such improvements demonstrate that explicitly optimizing the combination of feature blocks is substantially more effective than relying on raw concatenation, suggesting that the discriminative patterns are distributed across layers in a highly unbalanced manner. It also showed an improvement of 5% over concatenating features from all 12 layers.

At the cellular level, like the image-level analysis, we evaluated the performance of each feature block extracted from the 12 CNNs individually, repeating this process for the segmented nucleus and the annular region incorporating the nuclear membrane. As mentioned above, only the green channel was considered, and only one cell per image was selected for this initial step.

For the nucleus region, the training time for the OAO configuration averaged around 5 min, while OAA required approximately 14 min. Conversely, for the nuclear membrane region, the training time increased slightly, with the OAO configuration averaging around 6 min and OAA requiring approximately 15 min. At the cellular level, the OAO scheme once again proved to be both faster and more effective than the OAA configuration. The best performance at this level was achieved when focusing on the nucleus region rather than the annular region, incorporating the nuclear membrane. The superior performance of the nucleus region reinforces the biological expectation that most class-specific signals are localized within nuclear patterns, while the membrane contributes more weakly and with higher variability. However, our objective is to identify a feature concatenation strategy that can further enhance classification performance. To this end, we applied the GA also at the cellular level to determine the sub-optimal combination of features from different layers. In this case, we initialized the population with randomly generated individuals, ensuring diverse starting points for the optimization process. Consistent with the approach used at the image level, each individual in the GA was evaluated by the OAO method to compute the fitness. Starting from the initial population, the GA evolved for 25 generations, resulting in a total of 400 evaluations. The individual with the highest fitness comprises 4K features: 2K derived from the nucleus region and 2K from the nuclear membrane region. Table 7 summarizes the progression of results obtained at the cellular level. The best performing individual achieved an F1 macro score of 19.20%, reflecting an improvement of nearly 2% over the best result achieved using a single layer from the nuclear region and a gain of approximately 6% over the best result obtained using a single layer from the nuclear membrane region.

Additionally, it demonstrated a subtle improvement over the concatenation of features from the best layers derived from the nuclear region and the nuclear membrane region. To summarize the results obtained in the first phase, Table 8 shows the best results achieved at both the image and cell levels, considering well-known performance metrics. Overall, the first phase reveals a clear hierarchy in discriminative power: the green channel and nucleus region dominate, deeper CNNs generally perform better, but not uniformly, and selective feature integration via GA consistently outperforms naive concatenation. These findings provide strong guidance for the multi-label phase, where the search space becomes considerably more complex.

### 4.2. Multi-Class Multi-Label Results

To address the multi-class multi-label problem across the entire HPA database, we built upon the results obtained in the first phase using a subset of the HPA database. In this initial phase, we identified effective feature blocks extracted from CNNs at both the image and cell levels. We also constructed 276 classifiers using the OAO strategy at both the image and cell levels, resulting in a total of 552 binary SVM models. In this second phase, we applied a stacking strategy that leverages the combined knowledge of each binary classifier. This approach transforms their output into a compact and refined feature set, enabling the construction of a more robust and specialized second-level feature representation. In this second phase, at the cellular level, we segmented all cells in each image, excluding those located at the image borders or too small. For each segmented cell, we extracted 276 second-level features, then computed the average for each of these features across all cells in an image. This approach ensured that the feature vector for each image at the cellular level had a consistent size of 276 features. To analyze the results by combining the image-level and cell-level features, we simply concatenated the 276 image-level features with the 276 cell-level features for each image, resulting in a unified feature vector of 552 values. Feature extraction was carried out on the full HPA dataset comprising 31,072 samples. At the image level, the extraction process required less than 4.5 h on a standard computer. For each image, features were extracted from the resized 512 × 512 images. Initially, 11K features were computed using the layers of the CNNs across the green, red, yellow and RGB channels. These 11K features were subsequently input into the 276 binary SVMs, whose outputs formed the second-level feature vector, consisting of 276 features. At the cellular level, feature extraction required just over 6 days on the same machine. For each image, segmentation was performed to identify individual cells. Selected cells were extracted from the green channel of the full-size images, focusing on both the nuclear area and the nuclear membrane region. For each cell, a 4K feature vector was computed using the CNN layers. This feature vector was then processed through the 276 binary SVMs. Finally, a single second-level feature vector was created by averaging the outputs of all ROIs from the image. On average, feature extraction required approximately 0.51 s per image at the image level and nearly 18 s per image at the cellular level. The marked difference in feature extraction time between image and cellular levels highlights the computational cost of full cell-level analysis, emphasizing the importance of efficient feature selection and averaging strategies. After splitting the dataset with an 80–20% ratio between training and testing using the iterative stratification strategy (ensuring that the training samples from the first stage remained in the training set for the second stage) we applied the BR and LP multi-label classification strategies. For a comprehensive comparative analysis, we evaluated these strategies using four classifiers: SVM with a linear kernel, Logistic Regression, Decision Treend KNN. Training and testing for the BR and LP strategies, utilizing the four classifiers, were conducted in Python on Google Colab. The experiments leveraged the efficient and robust implementation provided by the sklearn library [39]. For linear kernel SVMs, the regularization parameter “C” was optimized using 5-fold cross-validation within the 80% of the data allocated for training. The optimization considered values 0.01, 0.10, 1.00, and 10.00. Once the optimal “C” value was determined, the entire 80% training dataset was used to build the final SVM models, which were then evaluated on the remaining 20% of the data reserved for testing. For all other SVM parameters and the remaining classifiers, default settings from the sklearn library were used. In the case of KNN, we experimented with different values of K, observing that the performance was highest at K = 1 and decreased as K increased. This drop in performance occurs because rare classes cannot be classified accurately when K exceeds the number of examples present in those classes. Consequently, we report the results for K = 1 and K = 3. The training time using the BR strategy ranges from approximately 2 min (when Logistic Regression is employed) to about 18 min (in the case of parameter tuning and use of SVMs). In contrast, with the LP strategy, the training time varies from roughly 4 min (again, with Logistic Regression being the fastest) to approximately 27 min (when parameter tuning and SVMs are utilized). Table 9 presents the multi-class multi-label results using the F1 macro figure of merit, evaluated at the image level, cellular level and the combination of the two levels. Notably, the SVM consistently achieves the best results across both BR and LP strategies compared to the other three classifiers. The consistent superiority of SVM across both BR and LP strategies confirms its robustness in handling the high-dimensional second-level features, while simpler classifiers, such as Decision Tree, are more sensitive to noise and feature redundancy. This holds true for the image level, cellular level and their concatenation. In contrast, the Decision Tree classifier generally delivers the lowest performance. The KNN classifier with K = 1 performs well in both BR and LP modes by associating the test samples with the closest training examples. Interestingly, the Logistic Regression classifier demonstrates improved performance when used in LP mode.

Table 10 summarizes the results obtained by evaluating the image level and cellular level separately. The table highlights the performance improvement achieved through the concatenation of the two levels, demonstrating the added value of integrating information from both perspectives. Comparison of the best multi-label classification results at the image and cellular levels, emphasizing the gains achieved by combining the two levels. The improvement obtained by concatenating image-level and cellular-level features demonstrates the complementarity of global and local information, highlighting the value of multi-resolution representation for multi-label classification.

Table A3 in Appendix A provides a detailed breakdown of the classification results across the 28 classes considered in the study. For each class, the precision, recall and F1-score metrics are reported, offering insights into the model’s ability to correctly predict samples for each label. In Table 11, we include only works that report multi-label results for all 28 classes of the HPA dataset. Comparing our results with those from other studies in the literature, the first notable observation pertains to the two rarest classes, “Rods & Rings” and “Microtubule Ends”. For these classes, our method achieves F1 scores of 0.8 and 0.4, respectively. As shown in Table 4 of [2], which summarizes the results for all 28 classes from the top teams in the Kaggle competition, most top teams reported F1 scores of zero for these two classes. Exceptions include the second-place team, which achieved an F1 score of 0.4 for “Rods & Rings” and the third-place team, which achieved 0.5 for “Microtubule Ends”. Similarly, the work in [12] reported an F1 score of zero for “Rods & Rings” and 0.33 for “Microtubule Ends”. Given the importance of accurately identifying rare classes, our method, which incorporates discriminative information at both the image level and the nucleus level by analyzing the nuclear region and the surrounding nuclear membrane, proves to be of significant interest. Accurately identifying rare classes like “Rods & Rings” and “Microtubule Ends” underscores the effectiveness of incorporating discriminative information from both image-level and nuclear-level features, which is often neglected in other studies. Unfortunately, for the other works listed in Table 11, results for all 28 individual classes are not available, making it impossible to determine their performance on these rare classes. Our work is particularly comparable to that of [12]. Like us, they applied a transfer learning strategy but focused on fine-tuning three well-known CNNs pre-trained on ImageNet (VGG16, ResNet152 and DenseNet169). After fine-tuning, they employed a stacked ensemble technique based on neural networks. In contrast, our approach utilizes transfer learning based on feature extraction, leveraging layers from pre-trained CNNs to feed SVM classifiers operating in OAO mode. The comparison with other works highlights that transfer learning combined with OAO-based feature extraction and stacking offers a scalable alternative to fine-tuning and neural network ensembles, while achieving comparable or superior results, particularly on rare classes. This strategy reduces dimensionality and generates second-level features. Another key difference is the choice of input channels: while ref. [12] used images composed of the red, blue and green channels, discarding the yellow channel, our GA based image-level analysis prioritizes features extracted from the green channel along with contributions from the red, yellow and RGB composite channels. Similarly, ref. [11] worked with RGB images constructed from the red, green and blue channels, whereas ref. [13] utilized the red, green and yellow channels. Overall, the multi-class multi-label phase confirms that integrating image and cell-level information, using SVM classifiers with optimized second-level features, and prioritizing discriminative channels substantially improves performance, particularly for challenging and rare classes. These insights can guide future improvements in large-scale protein localization tasks.

## 5. Conclusions

This study presents a sustainable transfer learning approach to address the complex challenges of high-imbalance, multi-class and multi-label classification in the HPA confocal microscopy dataset. The ability to identify automatically multiple and rare protein patterns is critical for biological research and clinical applications. The complexity of this task stems from three key factors: the significant class imbalance, especially for rare patterns; the presence of numerous patterns but also multi-label patterns within the same sample; and the spatial variation in protein expression across three distinct cellular regions such as the nucleus, nuclear membrane and cytoplasm. We adopt a green perspective leveraging well-known CNN architectures pre-trained on another large, complex and multi-class dataset. Instead of traditional fine-tuning, we use CNN layers as feature extractors coupled with SVM classifiers. We perform classification at both image and cell levels, incorporating nucleus and nuclear membrane segmentation, which enhances recognition of rare and critical patterns. At the image level, the green channel is most discriminative, but combining information from all channels further improves classification performance. By leveraging a two-phase strategy and feature block selection via a GA, our approach effectively addresses these challenges while maintaining computational efficiency. In the first phase, we focused on multi-class, single-label classification, identifying optimal feature blocks and classification models using a reduced dataset. This phase established a robust foundation for scaling to multi-label classification in the second phase, where BR and LP strategies were applied. A stacking-based approach was used in this phase, where second-level features were generated from the outputs of binary classifiers using a OAO strategy. This enabled the creation of compact, refined feature sets tailored to the requirements of multi-label classification. By reducing dimensionality while retaining discriminatory power, our approach minimized computational costs, avoiding the need for extensive computational resources or dataset augmentation. Although stacking adds an additional layer of complexity, it is far less resource-intensive than fine-tuning deep CNNs from scratch. Using straightforward strategies and classifiers, we achieved an F1 macro score of 0.59 and a weighted F1 score of 0.68, results that are competitive with existing methods in the literature. In particular, we improved F1 scores for rare patterns, such as 0.8 for ‘Rods and Rings’ and 0.4 for ‘Microtubule Ends’, surpassing most previous work. Our findings highlight the utility of combining feature blocks from multiple CNN architectures to enhance classification performance, confirming our hypothesis that diverse architectural representations provide complementary information. Moreover, the GA feature block selection streamlined computational demands, demonstrating its potential as a robust tool for optimizing high-dimensional classification tasks. Additionally, our study intentionally did not incorporate data augmentation methods to establish a clear and computationally efficient baseline. Nonetheless, our approach has some limitations. In particular, we did not employ data augmentation or explore intermediate CNN layers, which could further enhance model generalization and discriminative power. These aspects are addressed as potential directions in the following Future Work section.

### Future Work

Despite its effectiveness, several avenues could further enhance the applicability and performance of our approach. One direction involves exploring the potential of extracting features from intermediate layers of CNN architectures, rather than only relying on the final layer. This approach could uncover additional discriminative power, contributing to improved classification performance. At the cell level, a clustering-based strategy [40] could be employed to group similar cells and analyze their traits across all segmented ROIs. Such an approach would be particularly beneficial for multi-label images where cells exhibit diverse characteristics [2]. To further enhance the feature extraction capabilities of our model, a promising direction is to explore the applicability and performance of more modern architectures, such as Vision Transformers (ViT). Additionally, systematically evaluating data augmentation and image preprocessing techniques could improve model generalization, particularly for rare classes, while providing a meaningful comparison with our established baseline. Another interesting direction is to explore hierarchical classification strategies, which can reduce the number of binary classifiers while maintaining performance, as suggested in [41]. Finally, a detailed qualitative error analysis and an investigation into Explainable AI (XAI) could offer insights into model behavior and suggest potential improvements. Finally, extending this methodology to other complex datasets would validate its adaptability and confirm its potential applicability beyond the domain of medical imaging.

## Figures and Tables

**Table 1:** Summary of representative studies on the HPA dataset, outlining their core methodologies and the scope of their analysis.

Reference	Core Methodology and Focus	Scope of Analysis
Ouyang et al. [2]	Optimized DenseNet architecture; ensemble of whole-image and cell-segmented pipelines; Multi-Label Stratification; extensive data augmentation.	Full dataset (28 classes), multi-label classification.
Wang et al. [5]	Ensemble of pre-trained CNNs (ResNet-50, DenseNet-121, SE-ResNeXt-50); composite Focal-Lovász loss for minority classes; 4-channel input.	Full dataset (28 classes), multi-label.
Al-Joudi et al. [6]	CNN-based approach (GapNet-PL), utilizing oversampling/undersampling for imbalance. Image-level analysis (4 channels).	Full dataset (28 classes), multi-label.
Sullivan et al. [7]	Loc-CAT deep model combined with large-scale citizen-science annotations; transfer learning from game-generated labels.	29 localization patterns; 33 M human annotations.
Zhang et al. [8]	Multi-Instance Multi-Label Learning (MIML). Features extracted from segmented cell patches (U-Net). Transfer Learning (ResNet-50, DenseNet-121).	Subset of 8 patterns; 5772 images.
Liimatainen et al. [9]	Custom CNN and FCN architectures (10 convolutional layers); 4-channel input; straightforward architecture design.	Subset of 13 classes; 20,000 images.
Tr et al. [10]	Hybrid Xception CNN compared against Conventional Handcrafted Features (Haralick, LBP, Zernike). Image-level analysis.	Subset of 15 classes; 14,094 samples.
Tu et al. [11]	Two-stage method: Self-Supervised Pre-training followed by Supervised Learning. MIML-like approach using patches. Used RGB Images.	19,777 Images (173,594 patches).
Aggarwal et al. [12]	Focuses on Transfer Learning (VGG16, ResNet152, DenseNet169) and a Stacked Ensemble. Uses 3 channels (Red, Blue, Green composite).	Full dataset (28 classes), multi-label.
Rana et al. [13]	Oversampling via non-linear mix-up and transformations to generate synthetic samples for imbalance. Image-level analysis. Uses 3 Channels (Red, Green, Yellow).	Full dataset (28 classes), multi-label.

**Table 2:** Selected well-known CNNs pre-trained on Imagenet.

Pre-Trained CNNs	Layers	Pre-Trained CNNs	Layers
AlexNet [16]	25	GoogleNet [17]	144
VGG-16 [18]	41	ResNet-50 [19]	177
VGG-19 [18]	47	Inception-v3 [20]	315
MobileNet-v2 [21]	53	ResNet-101 [19]	347
SqueezeNet [22]	68	DenseNet-201 [23]	708
ResNet-18 [19]	68	InceptionResNet-v2 [24]	824

**Table 3:** Pre-trained CNN architectures and input image sizes.

Pre-Trained CNNs	Layers
MobileNet-v2, ResNet-18, ResNet-50, ResNet-101, DenseNet-201, GoogleNet, VGG-16, VGG-19	224 × 224 × 3
AlexNet, SqueezeNet	227 × 227 × 3
Inception-v3, InceptionResNet-v2	299 × 299 × 3

**Table 4:** CNN and their respective layer used to features extraction.

Pre-Trained CNNs	Layer Used
GoogleNet	Loss3-classifier
MobileNet-v2	Logits
ResNet-18, ResNet-50, ResNet-101, DenseNet-201	Fc1000
AlexNet, VGG-16, VGG-19	Fc8
SqueezeNet	Pool10
Inception-v3, InceptionResNet-v2	Predictions

**Table 5:** Green channel concatenation of features from different layers at the image level. Best value in bold.

Features Concatenation	F1 Macro (OAO)	F1 Macro (OAA)
Best layer	0.2736	0.2664
Best two layers	0.3014	0.2837
Best three layers	0.3085	0.2879
Best four layers	0.3116	0.3085
Best five layers	**0.3201**	0.3083
Best six layers	0.3179	0.3027
All twelve layers	0.3155	0.2940

**Table 6:** Result comparison at the image level. Best value in bold.

Configuration	F1 Macro
Best layer green channel	0.2736
Best five-layer green channel	0.3201
All twelve-layer green channels	0.3155
Best individual from AG (green channel)	0.3220
Best individual from AG	**0.3681**

**Table 7:** Green channel concatenation of features from different layers at the cellular level. Best value in bold.

Configuration	F1 Macro
Best layer nuclear region	0.1699
Best layer nuclear membrane region	0.1301
Best layers concatenation from two region	0.1865
Best individual from GA	**0.1920**

**Table 8:** Best results comparison at the image and cellular levels.

	F1 (Macro)	Precision (Macro)	Recall (Macro)	F1 (Weighted)
Image Level	0.37	0.35	0.52	0.48
Cellular Level	0.19	0.21	0.26	0.34

**Table 9:** Multi-label classification results for image-level, cellular-level and combined levels. Best value in bold.

	Image Level (F1 Macro)	Cell Level (F1 Macro)	Image & Cell (F1 Macro)
BR (logistic)	0.3288	0.2624	0.4116
BR (decision tree)	0.3179	0.2831	0.3388
BR (Knn k = 1)	0.4394	0.3454	0.4608
BR (Knn k = 3)	0.3333	0.2831	0.3719
BR (SVM)	**0.4553**	**0.3566**	**0.5269**
LP (logistic)	0.4533	0.2994	0.3037
LP (decision tree)	0.2348	0.2188	0.2557
LP (Knn k = 1)	0.4553	0.3416	0.5269
LP (knn k = 3)	0.4081	0.3319	0.4405
LP (SVM)	**0.5008**	**0.4231**	**0.5909**

**Table 10:** Best results multi-label classification at the image, cellular and combined two levels. Best value in bold.

	F1 (Macro)	Precision (Macro)	Recall (Macro)	F1 (Weighted)
Image Level	0.50	0.62	0.44	0.59
Cellular Level	0.42	0.49	0.38	0.60
Concatenation Image & Cell	**0.59**	**0.64**	**0.56**	**0.68**

**Table 11:** Comparison of state-of-the-art methods on the HPA dataset. Best value in bold.

	F1 (Macro)	Precision (Macro)	Recall (Macro)	F1 (Weighted)
**Human level** [2]	**0.71**	**-**	**-**	**-**
Winner Kaggle competition [2]	**0.59**	**0.67**	**0.55**	**-**
Wang et al. [5]	0.53	-	-	-
Rana et al. [13]	0.48	-	-	-
Tu et al. [11]	0.40	0.67	0.35	0.72
Sullivan et al. [7]	0.47	-	-	-
Al-Joudi et al. [6]	-	-	-	0.54
Aggarwal et al. [12]	0.56	0.63	0.53	0.71
**Our approach**	**0.59**	**0.64**	**0.56**	**0.68**

## Data Availability

Data are contained within the article.

## Appendix A

**Table A1:** Patterns name of multi-class HPA dataset.

	Pattern Name		Pattern Name
0	Nucleoplasm	14	Microtubules
1	Nuclear Membrane	15	Microtubule Ends
2	Nucleoli	16	Cytokinetic Bridge
3	Nucleoli Fibrillar Center	17	Mitotic Spindle
4	Nuclear Speckles	18	Microtubule Organ. Centre
5	Nuclear Bodies	19	Centrosome
6	Endoplasmic Reticulum	20	Lipid Droplets
7	Golgi Apparatus	21	Cell Junctions
8	Peroxisomes	22	Plasma Membrane
9	Endosomes	23	Mitochondria
10	Lysosomes	24	Aggresome
11	Intermediate Filaments	25	Cytosol
12	Actin Filaments	26	Cytoplasmic Bodies
13	Focal Adhesion Sites	27	Rods & Rings

**Table A2:** Average feature extraction time for CNN architectures.

Pre-Trained CNNs	Layers	Layer for Feat. Extraction	Mean Time for One Image (Seconds)
AlexNet	25	Fc8	0.027
VGG-16	41	Fc8	0.304
VGG-19	47	Fc8	0.348
MobileNet-v2	53	Logits	0.055
SqueezeNet	68	Pool10	0.042
ResNet-18	68	Fc1000	0.065
GoogleNet	144	Loss3-classifier	0.114
ResNet-50	177	Fc1000	0.095
Inception-v3	315	Predictions	0.188
ResNet-101	347	Fc1000	0.172
DenseNet-201	708	Fc1000	0.255
InceptionResNet-v2	824	Predictions	0.367

**Table A3:** Performance metrics for each of the 28 classes with the concatenation of image and cell level.

Pattern Name		F1	Precision	Recal
Nucleoplasm	0	0.85	0.84	0.86
Nuclear Membr.	1	0.79	0.83	0.75
Nucleoli	2	0.65	0.70	0.62
Nucleoli Fibr. Cen.	3	0.62	0.67	0.58
Nuclear Speckles	4	0.75	0.80	0.70
Nuclear Bodies	5	0.58	0.66	0.52
Endoplasmic Ret.	6	0.53	0.57	0.50
Golgi Apparatus	7	0.59	0.63	0.55
Peroxisomes	8	0.70	0.78	0.64
Endosomes	9	0.70	0.64	0.78
Lysosomes	10	0.67	0.56	0.83
Intermediate Fil.	11	0.63	0.68	0.59
Actin Filaments	12	0.56	0.67	0.48
Focal Adhes. Sites	13	0.51	0.62	0.43
Microtubules	14	0.71	0.75	0.68
Microtubule Ends	15	0.40	0.40	0.40
Cytokinetic Bridge	16	0.30	0.52	0.21
Mitotic Spindle	17	0.46	0.51	0.42
Microt.Org. Cen.	18	0.45	0.53	0.39
Centrosome	19	0.37	0.48	0.30
Lipid Droplets	20	0.57	0.62	0.53
Cell Junctions	21	0.61	0.65	0.58
Plasma Membrane	22	0.47	0.57	0.40
Mitochondria	23	0.71	0.74	0.69
Aggresome	24	0.45	0.65	0.34
Cytosol	25	0.67	0.67	0.68
Cytoplasmic Bod.	26	0.44	0.56	0.36
Rods & Rings	27	0.80	0.67	1.00
Macro AVG		0.59	0.64	0.56
Weighted AVG		0.68	0.71	0.66
